# Retraction: Development of Gene-Pyramid Lines of the Elite Restorer Line, RPHR-1005 Possessing Durable Bacterial Blight and Blast Resistance

**DOI:** 10.3389/fpls.2019.00125

**Published:** 2019-02-06

**Authors:** Frontiers Editorial Office

Following publication, the publisher was alerted to potential image manipulation and duplication in this paper. An investigation was initiated, in accordance with our established procedures, which revealed image duplication in Figure 4 of the article. The publisher requested the original images from the authors, and an examination of those images revealed computer manipulation. Given extant concerns over the validity of the study, the article has been retracted.

The retraction of the article was approved by the Field and Specialty Chief Editors of Frontiers in Plant Science.

